# Pre- and Post-Operative Relationship between Radiological Measures and Clinical Outcomes in Women with Hallux Valgus

**DOI:** 10.3390/jcm11133626

**Published:** 2022-06-23

**Authors:** Luci M. Motta, Ignacio Manchado, Gustavo Blanco, Felipe García-Flemate, Jesús González, Gerardo L. Garcés

**Affiliations:** 1 Hospital Perpetuo Socorro, 35007 Las Palmas, Spain; luci.motta@traumaquir.es (L.M.M.); nachomanchado@gmail.com (I.M.); gustblan@gmail.com (G.B.); fegarf@gmail.com (F.G.-F.); 2 Departamento de Ciencias Médicas y Quirúrgicas, University of Las Palmas de Gran Canaria, 35016 Las Palmas, Spain; 3 Unidad de Investigación, Hospital Dr Negrin, 35007 Las Palmas, Spain; josu.glez32@gmail.com

**Keywords:** hallux valgus, surgical correction, radiological measurements, clinical outcomes

## Abstract

The surgical correction of a hallux valgus (HV) deformity improves radiological parameters and clinical outcomes. However, it is not known how these improvements are related between themselves. In this retrospective study, 73 women were assessed preoperatively and 60 months after HV surgical correction. Several radiological parameters were measured: the hallux valgus angle (HVA), I–II intermetatarsal angle (IMA) and sesamoid position. The functional outcomes were assessed using the American Orthopaedic Foot and Ankle Society (AOFAS) Hallux Metatarsophalangeal-Interphalangeal (HMI) scale, and patient-reported outcomes (PROMs) were recorded with the Manchester–Oxford Foot Questionnaire (MOXFQ). A pre–post-surgery comparison of radiological and clinical values was performed, the correlation among them was studied and the differences pre–post-surgery in the radiological measurements compared with those for the clinical outcomes were studied. The results show that all the radiological parameters, functional outcomes and PROMs improved significantly from their pre-operative values to the follow-up values. Multivariate regression analysis showed a significant relationship (*p* < 0.001) between the differential pre–post-surgery AOFAS scoring only with two sesamoid position differential pre–post-surgery measures: position of medial sesamoid (PMS) and translation of the first metatarsal head (TMH). However, no significant association was observed between the pre–post-surgery radiological differences and the pre–post-surgery MOXFQ scoring.

## 1. Introduction

There are many surgical techniques for correcting hallux valgus deformities. The preoperative assessment includes an exhaustive clinical examination of both feet of the patient and a weight-bearing anteroposterior (AP) and lateral radiograph. This allows the assessment of the type and grade of the deformities to be corrected and the results to be compared to postoperative radiographies. The parameters most used for the radiological evaluation are the hallux valgus angle (HVA), the first-to-second intermetatarsal angle (IMA) and the position of the sesamoids [1].

Sesamoid subluxation off the head of the first metatarsal is indicative of an HV deformity. Currently, it is considered that during the progression of an HV deformity, the head of the first metatarsal bone drifts medially away from the sesamoids, whereas the sesamoids retain their anatomical relationship to the second metatarsus [2]. The sesamoid position is assessed through several radiological parameters [3,4]. The classifications most frequently used to assess the tibial sesamoid position (TSP), which represents the position of the medial sesamoid with respect to the axis of the first metatarsal bone, are the method of Hardy and Clapham [5], which classifies seven grades, and the American Orthopaedic Foot Ankle Society classification [6], with only four possible positions. The distance of the fibular sesamoid (DFS) between the axis of the second metatarsus and the lateral sesamoid is obtained [3]. The translation of the first metatarsal head (TMH) between the axis of the second metatarsal bone and the head of the first metatarsus is measured [3].

In addition to the HVA and IMA, many surgeons aim to correct the sesamoid position when surgically treating an HV deformity. Chen et al. [7] found that the TSP improved from Grade VII preoperatively to Grade IV postoperatively (Hardy and Clapham classification [5]), two years after surgery. However, some authors have shown that first metatarsal osteotomies can improve head-to-sesamoid congruency, but the sesamoids’ position remains unchanged with respect to the second metatarsal bone [3,8,9,10,11].

Several authors have outlined the importance of the sesamoid position for the recurrence of the deformity [3,12]. The assessment of the lateral sesamoid position (LSP) in relation to the flare of the first metatarsal head on a weight-bearing AP radiograph should help clinicians to grade the severity of the HV deformity [2]. Okuda et al. [13] concluded that the incomplete reduction of the sesamoids following corrective surgery resulted in a higher recurrence of hallux valgus 41 months after the surgery. Hagio et al. [14] found that a Grade ≥ 2 sesamoid position was significantly associated with the recurrence of the deformity. Ezzatvar et al. [12], in a systematic review, observed that a post-operative TSP ≥ 4 was strongly correlated with the recurrence of the deformity. However, there is some controversy about the influence of the pre-operative sesamoid position on the hallux valgus deformity. Machado et al. [4] observed that neither the absolute nor the relative distance of the lateral sesamoid bone to the second metatarsus was different between a hallux valgus group (HVA > 15°) and a control group of patients (HVA < 15°). Kaufman et al. [15] found no significant correlation between the pre-operative sesamoid position and the recurrence of an HV deformity after surgical correction. On the other hand, some authors have reported that the pre-operative sesamoid position significantly affected recurrence years after the surgical correction of an HV [16,17].

The influence of the sesamoid position on the clinical outcome of an HV deformity is another source of controversy. Wilson et al. [18] reviewed 46 patients who underwent scarf osteotomy and concluded that, although patient satisfaction was dependent on the HVA and IMA, there was little correlation between the change in sesamoid position and patient satisfaction. Zitouna et al. [19] observed a significant increase in AOFAS score from before to one year after the operation. However, there was no relationship between the post-operative sesamoid position and clinical outcome. Contrarily, Chen et al. [7] found that subjective and functional scores were significantly better in patients with “normal” TSP in comparison with “outliers”. Mathews et al. [20] observed that no radiographic variable showed even a moderate correlation with any of the Foot and Ankle Outcome Score (FAOS) subscales, with the exception of TSP in patients aged 56 years and older.

The main objective of the surgical correction of HV is to correct the hallux alignment, improving the functional and patient-reported outcomes. Since the relationship among them is not clear, the main purpose of this work was to study the correlation of the differences between the pre- and post-operation values of the radiological parameters, mainly the sesamoid position, with the differences between the preoperative vs. at follow-up results of the clinical outcome scores.

The specific objectives were as follows:

1. To compare the preoperative vs. post-operative follow-up values of the radiological parameters and clinical outcomes related to HV deformities.
2. To study the correlation of the pre- and post-operative radiological values with the pre- and post-operative values of functional and patient-reported outcome scores.

Our hypothesis was that the improvement in radiological parameters after the operation should be significantly related to the improvement in clinical score results.

## 2. Materials and Methods

This was a retrospective study of 73 female patients who underwent an operation to correct unilateral hallux valgus deformities (angle > 20°) undertaken by the same surgical team between 2013 and 2014. Their median age was 57 (IQR: 46–63; range: 22–77). Informed consent was obtained from the patients to participate in the study. This work was approved by the Ethic Local Committees (Ref CEIH-2018-02 and CEIm-LP-2021-418-1) and carried out according to the principles of the Helsinki Declaration.

Patients were included in the study if they were women older than 18 years with a unilateral hallux valgus angle (HVA) >20° that caused some kind of discomfort or pain during activities of daily living and who agreed to participate in the study. The exclusion criteria were bilateral symptomatic hallux valgus; a previous operation or fracture on the affected foot; hallux rigidus; general or local inflammatory, neurological or vascular disease; and lesser toe deformities on the same foot subsidiaries of surgical correction. An asymptomatic contralateral hallux valgus was not an exclusion criterion.

### 2.1. Type of Operation

**Figure 1:**
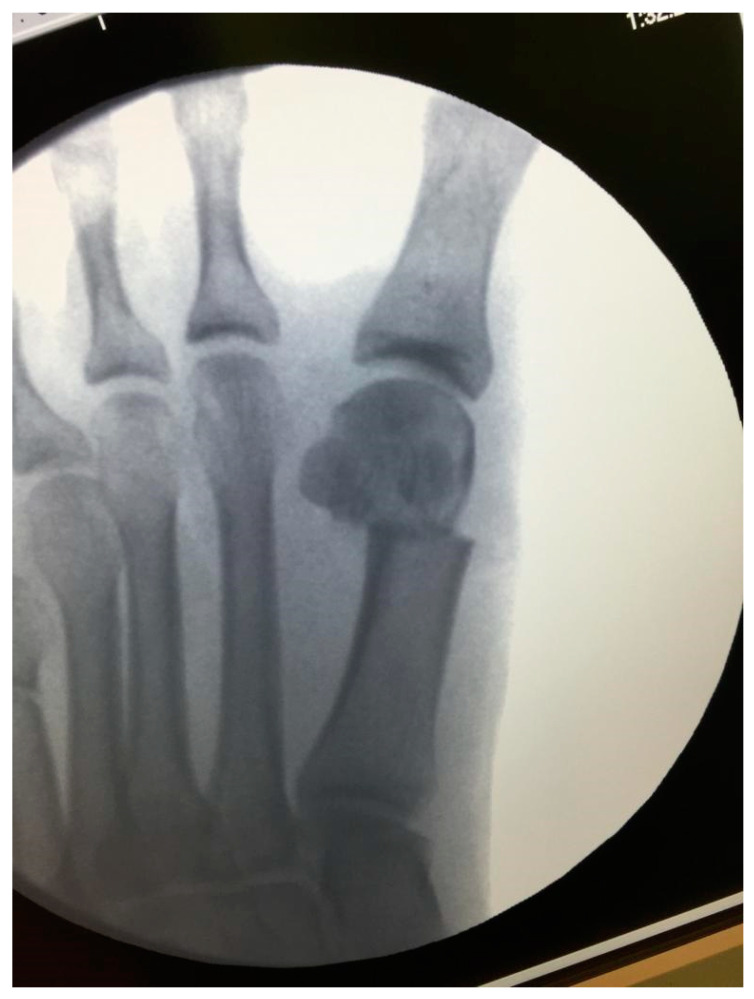
Fluoroscopic view of metatarsal metaphyseal osteotomy with lateral head displacement.

The operations were executed using a minimally invasive technique modified from the Isham–Reverdin procedure [21]. This technique has been exhaustively described by Biz et al. [22]. In summary, under local anesthesia and sedation, using fluoroscopy vision, 3 percutaneous incisions of 3–4 mm were made. The first one was at the level of the first metatarsal neck, to allow a bunionectomy and a transverse osteotomy of the metatarsal bone, just proximal to the sesamoid level, to be performed. Contrarily to Biz et al.’s technique [22], we pushed out the metatarsal head to translate it laterally approximately one-fourth of the osteotomy line length, closing the inter-metatarsal space (Figure 1). Through a second dorsal incision, immediately lateral to the metatarsophalangeal joint, the lateral soft tissues and the transverse head of the abductor hallucis were released to allow the translation of the first metatarsal head. In some cases, through a third incision, an incomplete medial transverse osteotomy of the proximal phalange (Akin osteotomy) was made 1 cm distal to the articular line to improve the hallux valgus angle. No internal fixation was carried out. The correction was kept with a dressing around the hallux, under fluoroscopic vision. Immediate weight bearing was allowed postoperatively, using a shoe with a flat, stiff sole, and the dressing was changed every two weeks over 8 weeks after the operation.

### 2.2. Assessment Method

Scores on the American Orthopaedic Foot and Ankle Society (AOFAS) Hallux Metatarsophalangeal-Interphalangeal (HMI) scale [23,24] and the Manchester–Oxford Foot Questionnaire (MOXFQ) [25] and radiographies were obtained from the patients 4–5 days pre-operation and at follow-up, after a median of 60 months (IQR: 51–82).

### 2.3. Radiographic Measurements

**Figure 2:**
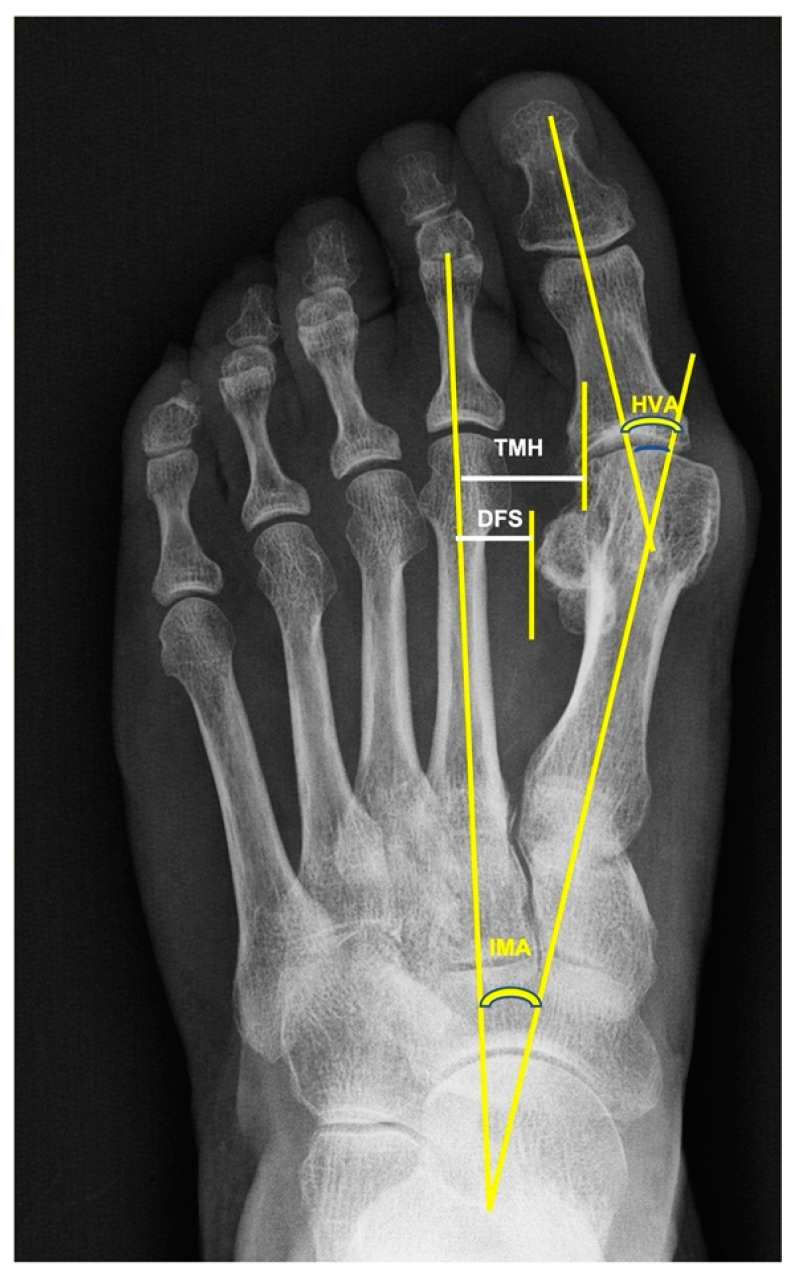
Radiological measures. **DFS:** Distance of the fibular sesamoid bone to the second metatarsal axis. **TMH:** Translation of the first distal metatarsal head. **HVA:** Hallux valgus angle. **IMA:** I–II intermetatarsal angle.

Weight-bearing antero-posterior and lateral radiographies of both feet were obtained preoperatively and at follow-up. All the radiographs were obtained using a picture-archiving and communications system (PACS; General Electronic, Chicago, IL, USA). The distances and angles were computed automatically using the Centricity Foot Print Universal viewer, version 6.0.SP 10.2.1 (GE Healthcare, Chicago, IL, USA), following Choi et al.’s [3] criteria (Figure 2). The hallux valgus angle (HVA) between the line connecting the center of the first metatarsal base and the center of the metatarsal head with the line connecting the centers of the proximal and distal articular surfaces of the proximal phalanx was measured. The translation of the first metatarsal head (TMH) was the distance between the second metatarsal axis and the lateral line of the first metatarsal head. The distance of the fibular sesamoid (DFS) was assessed by measuring the distance between the perpendicular line of the second metatarsal axis and the lateral margin of the fibular sesamoid. Measurements were taken twice by two researchers, and the mean was used for statistical analysis (the intra- and interobserver differences were less than 10%). The tibial sesamoid position (TSP) was determined using the 4 classification grades of the American Orthopaedic Foot Society [6]. On this scale, the medial sesamoid position is graduated according to its location with respect to the longitudinal axis of the first metatarsal bone. Grade 0 indicates that the tibial sesamoid is medial to this line; Grade 1 indicates that < 50% of its transversal diameter is overlapping the line; an overlap > 50% of its diameter is Grade 2; a lateral displacement of the complete sesamoid beyond that line is classified as Grade 3.

### 2.4. Statistical Analysis

The Shapiro–Wilk test was used to check the normality. The median (25–75% IQR) and mean (95% CI) values were used as the quantitative variables. Student’s t-test for paired samples was used to compare the differences between the mean results prior to the operation and at the final follow-up. Multiple linear regression was used to predict numerical variables. Due to the sample size, the linear regression coefficients were also calculated using the Bootstrapping technique, with 2000 repetitions. To check the multicollinearity of the predictive variables, the statistical variance inflation factor (VIF) was used. In all cases, its value was less than 5. Furthermore, *p*-values < 0.05 were considered significant. The data were analyzed with the R Core Team 2021 program, version 4.1.2 (R Foundation for Statistical Computing, Vienna, Austria).

## 3. Results

Thirty-five operations were on left feet, and 38 were on right ones. A few complications were encountered. Five patients had transfer metatarsalgia, with four of them being treated conservatively. One patient had postoperative hallux varus. Two patients were reoperated due to valgus recurrence; one more, for exostosis of the first metatarsus; and another one, for mallet finger. Two patients complained of persistent pain in the forefoot.

Table 1 shows the preoperative and final follow-up values of the quantitative variables. The results of all these variables were significantly improved at follow-up in comparison with the preoperative results (*p* < 0.001 for all cases). The median HVA decreased from 29.8° preoperatively to 10.3° at follow-up. The median IMA decreased from 13.3° preoperation to 8.6° at follow-up. The median DFS and TMH decreased from 11.99 and 18.17 mm preoperatively to 10.35 and 15.5 mm at follow-up, respectively. The median AOFAS score improved from 35 preoperatively to 90 at follow-up, while the median MOXFQ score improved from 29 before the operation to 18 at follow-up.

Table 2 shows the distribution of cases for each grade of TSP. No cases of Grade 0 were observed preoperatively, and 18 cases were present at follow-up. Grade 1 was observed in 16 cases preoperatively and in 40 cases at follow-up, Grade 2 in 30 cases preoperatively and in 15 cases at follow-up and Grade 3 in 27 cases preoperatively and no cases at follow-up. Individually, considering Grade 0 as the best situation, 1 case improved by three levels (from Grade 3 preoperatively to Grade 0 at follow-up), 22 cases improved by two levels, 41 cases improved by one level, 8 cases showed no change and 1 case was classified with a worse level.

The relationship of the preoperative AOFAS values (AOFAS Pre) with the radiological measures was not significant (Table 3).

Similarly, the relationship of the postoperative AOFAS values (AOFAS Post) with the radiological measures was also not significant (Table 4), although the IMA was nearly significant (*p* < 0.075), with a negative association (b = −2.28).

Differential pre-postoperative AOFAS scoring was significantly related to differential pre-postoperative values of PSM (negatively, b: −8.65) and THM (positively, b: 2.12) (*p* < 0.001 in both cases). However, the relationship with the rest of the radiological variables was not significant (Table 5).

The association of the MOXFQ preoperative values with the radiological measure values was non-significant (Table 6).

Similarly, the relationship between the post-operative values of the MOXFQ score and the post-operative values of the radiological parameters was non-significant (Table 7), as it was non-significant the association between the differential pre-postoperative values of the MOXFQ scores with the differential pre-postoperative values of the radiological measures (Table 8).

## 4. Discussion

Two findings should be outlined in our work. First, the radiological and clinical parameters improved significantly from preoperative values to follow-up. Second, the improvement of AOFAS scoring was only significantly associated with improvement in some parameters of the sesamoid position. However, the relationship between the improvement in MOXFQ scoring and the change in radiological measures was not significant.

There is evidence that females younger than 65 years with HV had a statistically significantly worse quality of life than females of the same age group in the general population [26], and that HV surgery resulted in decreased body pain and improved physical function and patient quality of life [27]. More than 100 different procedures for treating hallux valgus have been described [28]; they include combinations of soft tissue balancing, bone osteotomies and joint fusion. The aim of most operations is to obtain a hallux metatarsophalangeal angle of less than 15° and an I–II IMA of less than 10°. However, the importance of the sesamoid position in HV deformities is a subject of controversy. Some authors outlined that the lateral sesamoid position was not different between patients with hallux valgus (HVA > 15°) and a control group (HVA < 15°) [4].

The first point to be highlighted in this work is the fact that the HVA and IMA values, the variables most frequently used to quantify HV deformities, significantly improved from before the operation to the follow-up. This has also been frequently reported with the use of different surgical techniques [3,7,15,19,22,29,30,31]. In our work, two of the parameters used to assess the sesamoid position, namely, the DFS and TMH, also significatively improved from the pre- to the postoperative assessment. The preoperative vs. postoperative differential DFS of 1.3 mm is like that observed by others [3]. It is likely that this very small difference and the different methods used for measurement are the reasons for the lack of agreement regarding the role of the DFS in this condition. While some authors [2] consider there to be a strong relationship between the angular deformities and the lateral sesamoid position, others point out that the lateral sesamoid retains its relationship with the second metatarsal in the transverse plane and that the surgical correction of HV does not result in a medial shift or reduction in the sesamoid position [3,7,8,11]. The TMH in our patients improved by a mean of 3 mm from preoperation to the follow-up, which is smaller than the improvement observed by Choi et al. [3].

The radiological parameter most frequently used to assess the sesamoid position is the TSP. Most authors [7,10,29,32] have used the seven-grade Hardy and Clapham classification [5], while others [3,22] have used the simpler four-grade AOFS classification [6]. When comparing changes in TSP, all authors use the mean ± SD of the grade of all the patients preoperatively vs. at follow-up. However, since the TSP is an ordinal variable, we preferred to differentiate the pre–postoperative changes individually. Our findings show that the TSP improved by at least one grade at follow-up in 64 out of 73 cases, with 8 remaining unchanged and 1 getting worse. Improvements in TSP after surgery have also been reported by several authors, using different surgical techniques [3,7,8,9,10,15,29].

Our results show that the functional score, using the AOFAS-HMI scale, significantly improved from the preoperative assessment to the follow-up. This scale, with a 0–100 score, is the most frequently used functional outcome tool [27]. Other authors have also reported significant improvements in AOFAS scores after HV surgery using different techniques [3,11,19,22,29,30,31,33]. Our patients also showed a significant improvement in PROMs from the preoperative period to the follow-up, assessed through the MOXFQ. This is one of the best available tools for evaluating HV surgery outcomes and the most used [34]. It consists of 16 questions with a score out of 100 for three separate domains, although the three domain scores can be summarized into a single index score [35]. Similarly to our results, other authors have also reported significant improvements in MOXFQ scores after HV surgery [30].

Radiological parameters are the gold standard for assessing HV deformities pre- and postoperatively. Functional and patient-reported results provide complementary data for assessing the clinical situation and post-treatment outcomes. However, there is little information about the correlation between radiological results and clinical results in patients with HV. Some authors have reported a strong negative correlation of the HVA and IMA with the AOFAS score in non-operated patients [36]. Others found no correlation between these preoperative angles and AOFAS results [37]. Similarly, several authors have found that quality of life is decreased in patients with HV in comparison with a “normal” population, but the MOXFQ score showed no correlation with the radiological parameters [26,38]. Others have also observed that there is no correlation between radiological results and MOXFQ score after surgery [30]. Our results show that there is no significant relationship between the AOFAS and MOXFQ scoring with the HVA and IMA values both pre and postoperatively.

Many authors consider that the position of the sesamoids is very important in HV deformities, the ideal situation being both sesamoids being centered under the metatarsal head. As most authors focus their studies on the relationship of the sesamoid position with several radiological parameters, little attention has been paid to the clinical consequences of the changes in the sesamoid position after surgery. Chen et al. [7] found significantly better results for the AOFAS score when the TSP was <4 than when it was >5 (Hardy–Clapham classification). Zitouna et al. [19] observed that there was no relationship between the postoperative sesamoid position and the clinical outcome (AOFAS), regardless of the radiological classification used. Our results show that there was no significant association between the AOFAS or MOXFQ scores in the preoperative or the postoperative period with any of the radiological values of the sesamoid position in the same period. However, the differential pre to postoperative AOFAS values are significantly related to the PSM and TMH differential pre to postoperative values. We have not found references about the correlation between PROMs and the sesamoid position.

The main objective of our work was to study whether pre–post-surgery improvements in radiological parameters correlated with pre–post-surgery AOFAS and MOXFQ scores. Our results lead us to partially reject this hypothesis. To the best of our knowledge, this has not been studied previously. Despite advancements in operative techniques and the extraordinary number of procedures described for correcting hallux valgus (HV), there is still uncertainty as to why some patients thrive postoperatively whereas others do not. As others have also reported, our work shows a significant improvement in radiological and clinical outcomes after surgery. However, our findings lead us to assume that the correction achieved will not correlate to the clinical outcomes. Although the clinical importance of this lack of correlation cannot be determined from our work, we think that patients should be informed about it.

This work also has several limitations. Our population comprised only females, and we do not know if the results with men would have been different, although Choi et al. [3] observed no differences between the sexes regarding the clinical outcomes after chevron osteotomy. Our results are based only on one functional score and one PROM. However, the AOFAS and the MOXFQ are the most used questionnaires for assessing clinical results after HV surgery [27,34]. Our findings were observed after using a minimally invasive technique, and different results could be obtained with open surgical techniques. Nevertheless, currently, there is sufficient evidence for the reproducibility of the results and good clinical outcomes after the percutaneous correction of HV [22,26,28,29,31], and some authors have reported no differences in clinical results with open and mini-invasive techniques [39]. Another limitation of our study is that we did not consider how it was the relationship of radiological measures with specific important factors, such as the first ray mobility, which must be considered in routine clinical practice, before and post HV surgery [40,41].

## 5. Conclusions

All the radiological parameters, functional outcomes and PROMs improved significantly from their preoperative values to the follow-up values. However, apart from some sesamoid position values, no significant relationship between the pre–post-surgery radiological differences and the pre–post-surgery clinical outcome differences were found. Our findings allow us to assume that the correction achieved after surgery will not be correlated with the clinical outcomes, and this should be made known to the patients.

## Figures and Tables

**Table 1:** Preoperative and postoperative values of quantitative variables (*n* = 73). **Pre:** Preoperative values. **Post:** Values at follow-up. **DFS:** Distance of the fibular sesamoid bone to the second metatarsal axis. **TMH:** Translation of the first distal metatarsal head. **HVA:** Hallux valgus angle. **IMA:** I–II intermetatarsal angle. AOFAS: American Orthopaedic Foot and Ankle Society Questionnaire. **MOXFQ:** Manchester–Oxford Foot Questionnaire. Pre and Post data are medians (25–75% IQR). Mean Correction is the difference between the Pre and Post mean values (95% CI). Student’s *t*-test for paired samples was used.

	Pre	Post	Mean Correction (95% CI)	*p*-Value
**DFS**	11.99 (10.6–13.01)	10.35 (9.09–11.88)	1.29 (0.75–1.83)	**<0.001**
**TMH**	18.17 (16.69–20.46)	15.5 (13.78–17.46)	3.03 (2.32–3.75)	**<0.001**
**HVA**	29.8 (26–34)	10.3 (7.7–13.2)	20.23 (18.52–21.94)	**<0.001**
**IMA**	13.3 (12–15)	8.6 (7.8–9.6)	4.62 (4.06–5.17)	**<0.001**
**AOFAS**	35 (19–44)	90 (83–95)	54.4 (57.93–50.87)	**<0.001**
**MOXFQ**	29 (26–42)	18 (16–26)	11.22 (8.82–13.62)	**<0.001**

**Table 2:** Contingency table with number of cases and percentages of TSP classification, preoperatively (PRE) and at follow-up (POST). *p* < 0.001 in McNemar test. TSP: Tibial sesamoid position.

		TSP POST			
		Grade 0	Grade 1	Grade 2	Total
**TSP PRE**	Grade 1	9	6	1	16
		56.2%	37.5%	6.2%	21.9%
	Grade 2	8	20	2	30
		26.7%	66.7%	6.7%	41.1%
	Grade 3	1	14	12	27
		3.7%	51.9%	44.4%	37.0%
	Total	18	40	15	73

**Table 3:** Multivariate regression of preoperative AOFAS values with the preoperative radiological measures. **Pre:** Preoperative values. **DFS:** Distance of the fibular sesamoid bone to the second metatarsal axis. **TMH:** Translation of the first distal metatarsal head. **HVA:** Hallux valgus angle. **IMA:** I–II intermetatarsal angle. **TSP**: Tibial Sesamoid Position. AOFAS: American Orthopaedic Foot and Ankle Society Questionnaire.

Variables	Multivariate Regression					AM Bootstrapping	
	b	SE	B	IC (95%)	*p*	b	IC (95%)
Intercept	30.01	15.57	-	−1.08–61.1	0.058	29.24	−0.98–60.32
TSP_pre: Type 1	-	-	-	-	-	-	-
TSP_pre: Type 2	−1.15	4.15	−0.04	−9.44–7.14	0.782	−1.47	−9.99–7.19
TSP_pre: Type 3	−1.45	4.36	−0.06	−10.16–7.26	0.741	−1.73	−9.8–7.37
HVA_pre	−0.44	0.31	−0.19	−1.05–0.17	0.158	−0.45	−1.06–0.18
IMA_pre	0.72	0.74	0.12	−0.76–2.21	0.333	0.75	−0.67–2.19
DFS_pre	−0.14	0.69	−0.03	−1.53–1.24	0.838	−0.16	−1.5–1.41
TMH_pr	0.44	0.5	0.12	−0.56–1.44	0.38	0.48	−0.55–1.28
Adjusted R^2^	−0.04						

b: Regression coefficient. SE: Standart error. B: Standart coefficient. *p*: *p*-value.

**Table 4:** Multivariate regression analysis of postoperative AOFAS values with the postoperative radiological measures. **Pre:** Preoperative values. **DFS:** Distance of the fibular sesamoid bone to the second metatarsal axis. **TMH:** Translation of the first distal metatarsal head. **HVA:** Hallux valgus angle. **IMA:** I–II intermetatarsal angle. **TSP**: Tibial Sesamoid Position. AOFAS: American Orthopaedic Foot and Ankle Society Questionnaire.

Variables	Multivariate Regression					AM Bootstrapping	
	b	SE	B	IC (95%)	*p*	b	IC (95%)
Intercept	105.18	11.43	-	82.35–128.01	<000.1	105.59	82.23–131.61
TSP_post: Type 0	-	-	-	-	-	-	-
TSP_post: Type 1	0.76	3.5	0.03	−6.24–7.76	0.829	0.89	−4.6–7.91
TSP_post: Type 2	−0.88	5.08	−0.03	−11.03–9.27	0.863	−1.2	−11–8.32
HVA_post	−0.34	0.28	−0.16	−0.89–0.22	0.228	−0.31	−1.04–0.6
IMA_ post	−2.28	1.26	−0.24	−4.79–0.23	0.075	−2.44	−5.42–0.12
DFS_ post	−0.17	0.87	−0.03	−1.92–1.57	0.843	−0.31	−2.35–1.87
TMH_ post	0.36	0.71	0.09	−1.05–1.78	0.61	0.5	−1.01–2.17
Adjusted R^2^	−0.04						

b: Regression coefficient. SE: Standart error. B: Standart coefficient. *p*: *p*-value.

**Table 5:** Multivariate regression of pre-postoperative AOFAS difference values with the pre-postoperative differences of radiological measures. **Pre:** Preoperative values. **DFS:** Distance of the fibular sesamoid bone to the second metatarsal axis. **TMH:** Translation of the first distal metatarsal head. **HVA:** Hallux valgus angle. **IMA:** I–II intermetatarsal angle. **TSP**: Tibial Sesamoid Position. AOFAS: American Orthopaedic Foot and Ankle Society Questionnaire. b: Regression coefficient. SE: Standart error. B: Standart coefficient. *p*: *p*-value.

Variables	Multivariate Regression					AM Bootstrapping	
	b	SE	B	IC (95%)	*p*	b	IC (95%)
Intercept	51.48	5.53	-	40.44–62.52	<000.1	51.38	38.82–62.43
TSP_dif	−8.65	2.45	−0.4	−13.55–−3.76	<000.1	−8.72	−13.3–−3.18
HVA_d	−0.02	0.23	−0.01	−0.48–0.44	0.928	−0.01	−0.69–0.47
IMA_dif	0.17	0.7	0.03	−1.23–1.58	0.808	0.08	−1.41–1.52
DFS_dif	0.46	0.7	0.07	−0.94–1.86	0.511	0.38	−0.9–2.24
TMH_dif	2.12	0.56	0.43	1.01–3.23	<000.1	2.19	1.08–3.2
Adjusted R^2^	0.20						

**Table 6:** Multivariate regression of preoperative MOXFQ values with the preoperative radiological measures. **Pre:** Preoperative values. **DFS:** Distance of the fibular sesamoid bone to the second metatarsal axis. **TMH:** Translation of the first distal metatarsal head. **HVA:** Hallux valgus angle. **IMA:** I–II intermetatarsal angle. **TSP**: Tibial Sesamoid Position. MOXFQ: Manchester-Oxford Foot Questionnaire. b: Regression coefficient. SE: Standart error. B: Standart coefficient. *p*: *p*-value.

Variables	Multivariate Regression					AM Bootstrapping	
	b	SE	B	IC (95%)	*p*	b	IC (95%)
Intercept	21.24	12.31	-	−3.33–45.81	0.089	19.21	−9–43.59
TSP_pre: Type 1	-	-	-	-	-	-	-
TSP_pre: Type 2	4.26	3.28	0.2	−2.29–10.81	0.199	4.13	−2.43–10.07
TSP_pre: Type 3	−4.08	3.45	−0.19	−10.96–2.8	0.241	−4.33	−11.25–2.08
HVA_pre	−0.13	0.24	−0.07	−0.61–0.35	0.594	−0.14	−0.67–0.41
IMA_pre	0.3	0.59	0.06	−0.87–1.48	0.609	0.32	−0.76–1.37
DFS_pre	0.57	0.55	0.13	−0.53–1.66	0.305	0.65	−0.59–1.91
TMH_pre	0.27	0.39	0.09	−0.52–1.05	0.503	0.26	−0.5–1.17
Adjusted R^2^	0.04						

**Table 7:** Multivariate regression analysis of postoperative MOXFQ values with the postoperative radiological measures. **Post:** Postoperative values. **DFS:** Distance of the fibular sesamoid bone to the second metatarsal axis. **TMH:** Translation of the first distal metatarsal head. **HVA:** Hallux valgus angle. **IMA:** I–II intermetatarsal angle. **TSP**: Tibial Sesamoid Position. MOXFQ: Manchester-Oxford Foot Questionnaire. b: Regression coefficient. SE: Standart error. B: Standart coefficient. *p*: *p*-value.

Variables	Multivariate Regression					AM Bootstrapping	
	b	SE	B	IC (95%)	*p*	b	IC (95%)
Intercept	16.39	9.05	-	−1.67–34.45	0.075	16.14	−4.82–34.53
TSP_post: Type 0	-	-	-	-	-	-	-
TSP_post: Type 1	−3.03	2.77	−0.17	−8.57–2.51	0.278	−3.12	−7.95–1.43
TSP_post: Type 2	−3.81	4.02	−0.17	−11.84–4.22	0.347	−3.9	−10.59–1.71
HVA_post	0.35	0.22	0.21	−0.09–0.79	0.119	0.36	−0.17–1.09
IMA_ post	0.47	1	0.07	−1.51–2.46	0.636	0.39	−2.33–2.3
DFS_ post	0.02	0.69	0	−1.36–1.4	0.978	0.05	−1.52–1.66
TMH_ post	0.02	0.56	0.01	−1.1–1.14	0.973	0.03	−1.11–1.6
Adjusted R^2^	−0.02						

**Table 8:** Multivariate regression analysis of pre-postoperative MOXFQ values with the pre-postoperative radiological measures. **Dif:** Differential values. **DFS:** Distance of the fibular sesamoid bone to the second metatarsal axis. **TMH:** Translation of the first distal metatarsal head. **HVA:** Hallux valgus angle. **IMA:** I–II intermetatarsal angle. **TSP**: Tibial Sesamoid Position. MOXFQ: Manchester-Oxford Foot Questionnaire. b: Regression coefficient. SE: Standart error. B: Standart coefficient. *p*: *p*-value.

Variables	Multivariate Regression					AM Bootstrapping	
	b	SE	B	IC (95%)	*p*	b	IC (95%)
Intercept	−7.85	4.22	-	−16.27–0.58	0.067	−7.82	−17.04–1.06
TSP_dif	2.52	1.87	0.17	−1.21–6.25	0.182	2.51	−1.16–5.99
HVA_dif	0.04	0.18	0.03	−0.31–0.39	0.832	0.03	−0.29–0.35
IMA_dif	0.35	0.54	0.08	−0.72–1.42	0.513	0.37	−0.81–1.27
DFS_dif	−0.1	0.53	−0.02	−1.17–0.96	0.846	−0.09	−1.14–0.94
TMH_dif	−0.62	0.42	−0.18	−1.47–0.23	0.15	−0.65	−1.26–0.05
Adjusted R^2^	−0.02						

## Data Availability

The data that support the findings of this study are available from the corresponding author, G.L.G., upon reasonable request.
